# Karyotype characteristics and polymorphism peculiarities of *Chironomus bernensis* Wülker & Klötzli, 1973 (Diptera, Chironomidae) from the Central Caucasus and Ciscaucasia

**DOI:** 10.3897/CompCytogen.v9i3.4519

**Published:** 2015-06-23

**Authors:** Mukhamed Kh. Karmokov, Natalia V. Polukonova, Olga V. Sinichkina

**Affiliations:** 1 Federal state budget scientific establishment Tembotov Institute of Ecology of Mountain territories KBSC, RAS, I. Armand str., 37 a, Nalchik 360004, Russia; 2 Saratov State Medical University named after V.I. Razumovsky, Department of General Biology, Pharmacognosy and Botany, B. Kazach’ya str., 112, Saratov 410012, Russia

**Keywords:** Diptera, Chironomidae, *Chironomus bernensis*, polytene chromosomes, chromosome polymorphism, pericentromeric region, cytogenetic distances, larva premandible, Central Caucasus (northern macroslope), Ciscaucasia

## Abstract

Data about the karyotype characteristics, features of chromosomal polymorphism and larval morphology of populations of *Chironomus bernensis* Wülker & Klötzli, 1973 (Diptera, Chironomidae) from the Central Caucasus (the northern macroslope) and Ciscaucasia are presented. The characteristics of the pericentromeric regions of the long chromosomes of this species from Caucasian populations were very similar to the ones from some European populations (from Poland and Italy), but differed from Swiss and Siberian populations. In the North Caucasian populations 10 banding sequences were found: two in arms A, C, and E, and one in arms B, D, F, and G. Nine of them were already known for this species, and one, berC2, is described for the first time. Cytogenetic distances between all the studied populations of *Chironomus bernensis* show that close geographical location of all studied populations from the Central Caucasus and Ciscaucasia is reflected in their similar cytogenetic structure, but on the other hand, that they are more closely related to populations from Europe than to populations from Western Siberia. At the same time, all studied larvae from Caucasian populations have a four-bladed premandible, instead of a two-bladed one, as in the description of *Chironomus bernensis* from Switzerland (Wülker and Klötzli 1973, Polukonova 2005c). These peculiarities may indicate the relative isolation of the Caucasus from the viewpoint of microevolution. Further research on karyological and morphological characteristics of *Chironomus bernensis* from geographically distant regions is necessary as there is a possibility that the presently known species is actually polytypic and consists of several sibling species.

## Introduction

*Chironomus bernensis* was first described by Wülker and Klötzli in 1973 from Switzerland (Wülker and Klötzli 1973). The species belong to the “lacunarius” cytocomplex (2n=8, chromosome arm combinations AD, BC, EF, G).

The karyotype of *Chironomus bernensis* was studied early-on from Switzerland (Wülker and Klötzli 1973), Bulgaria, Poland, Northern Italy (Michailova 1989, Michailova et al. 2002, Petrova and Michailova 2002, Michailova et al. 2009) and Spain (Real et al. 2000). In Russia this species was known only from Western Siberia and the chromosomal polymorphism of those populations was described by Istomina and Kiknadze (Istomina and Kiknadze 2004, Kiknadze et al. 2007).

The aim of this work is to present the description of karyotype, chromosomal polymorphism and larval morphology of *Chironomus bernensis* from the Central Caucasus (the northern macroslope) and Ciscaucasia – Republic of Kabardino-Balkaria (RKB), Republic of North Ossetia-Alania (RNO-Alania), Karachai-Cherkess Republic (KCR) and Stavropol Krai. It was also important to compare characteristics of chromosomal polymorphism of *Chironomus bernensis* from Caucasus, Western Europe and Western Siberia.

## Methods

Fourth instar larvae were used in the karyological study. The larvae were collected from 12 sites of the Central Caucasus and Ciscaucasia: seven sites from Republic of Kabardino-Balkaria (RKB), one site from Republic of North Ossetia-Alania (RNO-Alania), one site from Karachai-Cherkess Republic (KCR), and four sites from Stavropol Krai (Table 1). In the aspect of the vertical zonation the site in KCR belongs to the Kuban variant, all sites in Stavropol Krai belong to the steppe zone and all sites in RKB and RNO-Alania belong to the Terek variant (typification of the zone variants are given according to Sokolov and Tembotov 1989).

**Table 1.** Collection sites and number of specimens of *Chironomus bernensis* of Central Caucasus.

Localities	Collection sites	Collection date	Number of specimens
RKB	43°27.05'N; 43°35.42'E, mouth of Nartia River, near Khasania village, altitude ca 440 m a.s.l.	21.12.07	3
	43°37.44'N; 43°55.09'E, main riverbed of Urvan River, near Koldrasynckyi hamlet, altitude ca 230 m a.s.l.	29.07.08	1
	43°22.59'N; 43°42.77'E, floodplain pool in riverbed of Kheu River, near Aushiger village, altitude ca 560 m a.s.l.	23.03.08	1
	43°29.16'N; 43°38.57'E, main riverbed of Nalchik River, Nalchik city, altitude ca 340 m a.s.l.	09.03.08	5
	43°45.02'N; 44°00.29'E, Prokhladnyi city, Vinzavod township, canal, altitude ca 200 m a.s.l.	18.02.09	1
	43°41.76'N; 44°00.39'E, former riverbed in mouth of Cherek River, near Oktyabrskyi village, altitude ca 210 m a.s.l.	21.03.10	9
	43°12.89'N; 43°39.37'E, 500 m over Zhemtala village, long-term waterbody, altitude ca 940 m a.s.l.	18.07.12	39
Stavropol Krai	43°58.71'N; 43°21.12'E, reservoir at Etoko River, in Verkhnetambukanskyi village, altitude ca 440 m a.s.l.	02.04.10	1
	44°42.72'N; 41°49.46'E, floodplain pool of Kuban River, near Kochubeevskaya village, altitude ca 280 m a.s.l.	14.10.10	2
	44°10.44'N; 42°40.81'E, floodplain pool of Kuma River, near Suvorovskyi village, altitude ca 450 m a.s.l.	14.10.10	4
	44°59.88'N; 41°45.33'E, Sengeleevskoe reservoir, near Sengeleevskaya village, altitude ca 230 m a.s.l.	15.10.10	1
RNO-Alania	43°19.85'N; 44°11.19'E, bed of lowered pond near Zmeiskaya village, altitude ca 310 m a.s.l.	05.05.10	1
KCR	44°21.82'N; 41°55.96'E, backwater in main riverbed of Malyi Zelenchuk River, near Adyl-Khalk village, altitude ca 420 m a.s.l.	14.10.10	17

In total 85 specimens of *Chironomus bernensis* were studied.

For karyotype analysis larvae were fixed in ethanol-glacial acetic acid (3:1). Slides of the chromosomes were prepared with ethanol-orcein technique (Dyomin and Ilyinskaya 1988, Dyomin and Shobanov 1990).

The identification of chromosome banding sequences for arms A, E and F was performed with use of photomaps of Wülker and Klotzli (1973) in the system of Keyl (Keyl 1962) and chromosome mapping for arms C and D was performed according to Istomina and Kiknadze (2004) in the system of Dévai et al. (Dévai et al. 1989). Microscope Carl Zeiss Axio Imager.A2 was used to study chromosome slides. Software packages PAST 2.17 and STATISTICA 10 were used for statistical analysis (cluster analysis).

The following parameters were used for comparison of characteristics of chromosomal polymorphism: the number of zygotic combinations, percentage of heterozygous larvae, number of heterozygous inversions per specimen, number of inversions per arm, number of banding sequences in a population.

Cytogenetic distances between populations were calculated according to Nei (Nei 1972).

## Results

**Figure 1.**
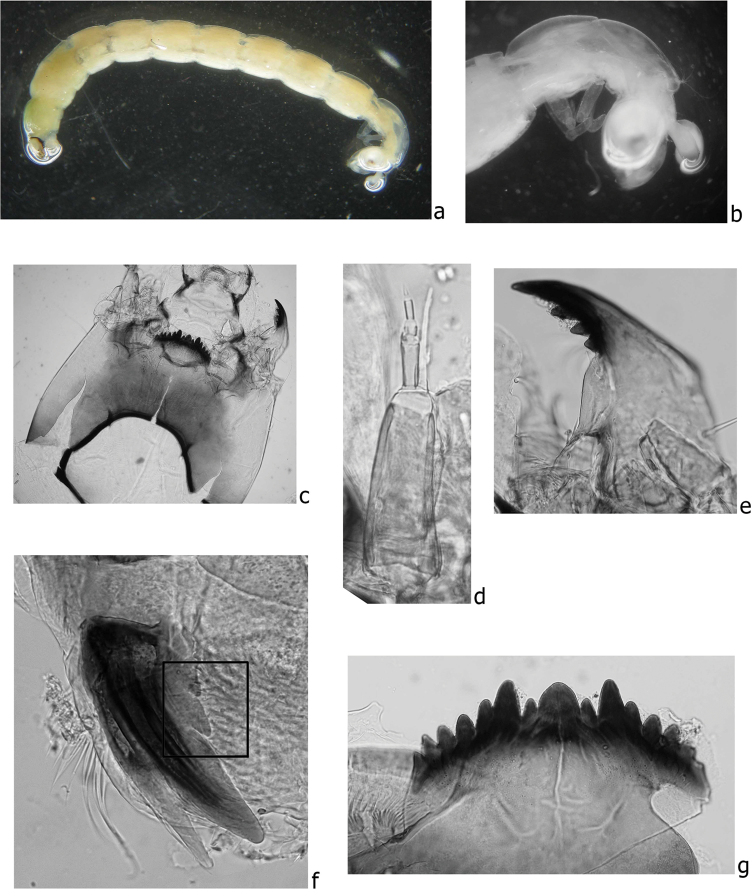
The larva of *Chironomus bernensis* from the Central Caucasus and Ciscaucasia, **a** total view **b** ventral tubuli at segment VIII **c** head ventrally **d** antenna **e** mandible **f** premandible with additional teeth marked in the square **g** mentum.

The larvae of the genus *Chironomus* Meigen, 1803 in all studied sites of the Central Caucasus and Ciscaucasia were attributed to *Chironomus bernensis* by chromosomal and morphological characteristics. Morphological characteristics are presented on Fig. 1a–g. In general, the larval characters of *Chironomus bernensis* from Caucasian sites are similar to those described previously for this species by Wülker and Klötzli (1973), however, some noticeable distinctions were found. Thus, it was stated by Wülker and Klötzli (1973) that larva of *Chironomus bernensis* was not different from that of *Chironomus commutatus*. Indeed, both species have the same type of larva (“bathophilus”), degree of gular sclerite pigmentation and structure of mentum and antenna. However, the fourth tooth of mandible of *Chironomus bernensis* from Caucasian populations was dark brown or dark (Fig. 1e), while it is pale brown in *Chironomus commutatus* according to Shobanov (2000). It is possible that Wülker and Klötzli (1973) did not notice this distinction. Another morphological peculiarity that was revealed was the presence of four-bladed premandibles in all studied larvae (Fig. 1f) instead of the two-bladed ones of *Chironomus commutatus* (Laville 1971, Polukonova 2005c). The exterior tooth of the premandible in *Chironomus bernensis* larvae of the North Caucasian populations was 2–2.5 times narrower than the inner one, longer and awl-shaped at the edge, the inner tooth was split into two small additional teeth near its basis (Fig. 1f).

### Karyotype of *Chironomus bernensis* from the Central Caucasus and Ciscaucasia

**Figure 2.**
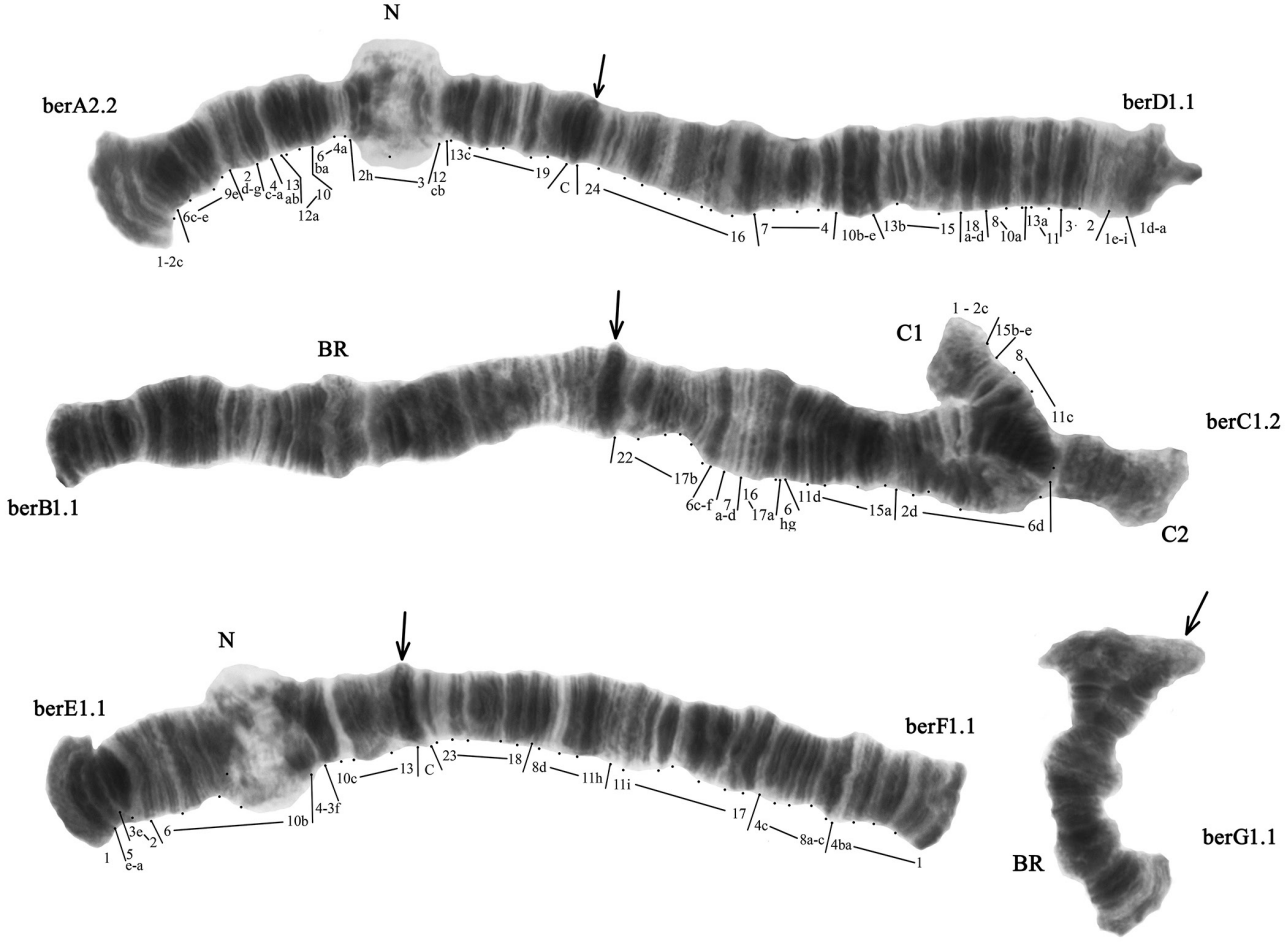
Karyotype of *Chironomus bernensis* Northern Caucasus. berA2.2, berD1.1 etc. – zygotic combinations of banding sequences; BR – Balbiani rings, N – nucleoli. Arrows indicate centromeric regions.

The diploid number of chromosomes in *Chironomus bernensis* karyotype is 2n=8, chromosome arm combination is AD, BC, EF, G – “lacunarius” cytocomplex (Fig. 2). Chromosomes AD and BC are metacentric, EF is submetacentric and G is telocentric. Two well developed nucleoli (N) are located on arms A and E. There are two Balbiani rings (BR) in the karyotype: one is situated in arm B and the other – in arm G, but in populations that we have studied the activity of both both BR was greatly reduced (Fig. 2).

The centromeric bands of long polytene chromosomes of *Chironomus bernensis* from the studied populations are large and belong to n-type (according to the classification by Shobanov (2002)). One of the peculiarities of the karyotype of *Chironomus bernensis*, as indicate before by Istomina and Kiknadze (2004), is comparatively large telomeres of all chromosomes that often results in a presence of ectopic pairing between different chromosomes. We also observed such ectopic pairing with very low frequency and without any clear pattern between arms B and D in some specimens from different collection sites of Caucasus.

### Banding sequences and chromosomal polymorphism of *Chironomus bernensis* from the Central Caucasus and Ciscaucasia

Up until now, 16 banding sequences have been described in the banding sequences pool of *Chironomus bernensis* (Table 2). In populations studied in this paper only 9 of those banding sequences were present, and one banding sequence has been found for the first time, so in total 10 banding sequences were found in Caucasian populations (Table 3).

**Table 2.** Catalog of banding sequences in the banding sequences pool of *Chironomus bernensis*.

**Arm**	**Sequence**	**Order of bands**	**Authors of mapping**
A	berA1	1-2c 10a-f 11-13ba 4a-c 2g-d 9e-6e-a-4d 2h-3i 12cb 13-19 C	Wülker and Klötzli 1973
	berA2	1-2c 6c-e-9e 2d-g 4a-c 13ab-11 10f-a 6ba-4d 2h-3i 12cb 13-19 C	-//-
B	berB1	Not mapped	-//-
C	berC1	1-2c 15b-e 8-11c 6b-2d 15a-11d 6gh 17a-16 7d-a 6f-c 17b-22 C	Istomina and Kiknadze 2004
	berC2	1-2c 4hi-6b 11c-8 15e-b 4g-a-2d 15a-11d 6gh 17a-16 7d-a 6f-c 17b-22 C	Original data
D	berD1	1a-d 1i-e 2-3 11-13a 10a-8 18d-a 15-13b 10b-e 4-7 16-17 18e-24 C	Istomina and Kiknadze 2004
E	berE1	1a-i 5e-a 3e-2 6-10b 4-3f 10c-13 C	Wülker and Klötzli 1973
	berE2	1a-i 5e-a 3e-2 6-10b 12-11 10g-c 3f-4h 13 C	Petrova and Michailova 2002
	berE3	1a-i 6ba 2-3a-e 5 6c-h-10b 4h-3f 10c-13 C	-//-
	berE4	1a-i 5e-a 3e-2 7d-6 7e10b 4-3f 10c-13 C	Istomina and Kiknadze 2004
F	berF1	1-4b 8c-4dc 17-12 11i-a-9f-c 8ed 18-23 C	Wülker and Klötzli 1973
	berF2	1-4b 8c-5d 11i-17 4c-5c 11h-10 9f-c 8ed 18-23 C	-//-
	berF3	1-4b 8c-4dc 11i-17 11h-8ed 18-23 C	Petrova and Michailova 2002
	berF4	1-4b 8c-5d 11i-15e 5a-4c 17d-15f 5bc 11h-10 9f-c 8ed 18-23 C	Istomina and Kiknadze 2004
G	berG1	1 2 3 4 7ba 6 5 7c-e	Petrova and Michailova 2002
	berG2	Not mapped	Istomina and Kiknadze 2004
	berG3	Not mapped	-//-

**Table 3.** Frequency of banding sequences in different populations of *Chironomus bernensis*.

Banding sequence	Populations					
	Western Europe		Central Caucasus			Western Siberia (Istomina and Kiknadze 2004) 60 larvae
	Switzerland (Wülker and Klötzli 1973) 446 larvae	Italy (Petrova and Michailova 2002) 14 larvae	RKB, former riverbed in mouth of Cherek River (original data) 9 larvae	RKB, near Zhemtala village, long-term pool (original data) 39 larvae	KCR, M. Zelenchuk River (original data) 17 larvae	
berA1	0,950	0,821	0,056	0,313	0,411	1,000
berA2	0,050	0,179	0,944	0,687	0,589	-
berB1	1,000	1,000	1,000	1,000	1,000	1,000
berC1	1,000	1,000	0,444	0,700	0,853	1,000
berC2	-	-	0,556	0,300	0,147	-
berD1	1,000	1,000	1,000	1,000	1,000	1,000
berE1	1,000	0,928	0,833	0,975	0,971	0,992
berE2	-	0,036	-	-	-	-
berE3	-	0,036	0,167	0,025	0,029	-
berE4	-	-	-	-	-	0,008
berF1	0,680	abs†	1,000	1,000	1,000	-
berF2	0,320	abs	-	-	-	0,992
berF3	-	0,036	-	-	-	-
berF4	-	-	-	-	-	0,008
berG1	1,000	1,000	1,000	1,000	1,000	0,350
berG2	-	-	-	-	-	0,592
berG3	-	-	-	-	-	0,058
Number of banding sequences in population	9	12	10	10	10	11

† abs - data are absent.

**Figure 3.**
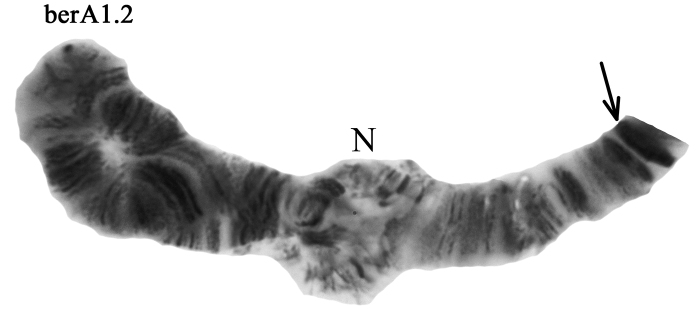
Heterozygous zygotic combination berA1.2. The designations are the same as in Fig. 2.

**Arm A.** Two banding sequences – berA1 and berA2 – were found in both homozygous and heterozygous state (Fig. 3, Table 2–4). Banding sequence berA2 in homozygote (berA2.2) was dominant in all populations studied (Table 3, 4).

**Table 4.** Frequency of zygotic combinations and parameters of chromosomal variability in different populations of *Chironomus bernensis*.

Zygotic combinations	Populations					
	Western Europe		Central Caucasus			Western Siberia (Istomina and Kiknadze 2004) 60 larvae
	Switzerland (Wülker and Klötzli 1973) 446 larvae	Italy (Petrova and Michailova 2002) 14 larvae	RKB, former riverbed in mouth of Cherek River (original data) 9 larvae	RKB, near Zhemtala village, long-term pool (original data) 39 larvae	KCR, M. Zelenchuk River (original data) 17 larvae	
berA1.1	0,889	0,643	-	0,025	0,235	1,000
berA1.2	0,101	0,357	0,111	0,617	0,353	-
berA2.2	-	-	0,889	0,358	0,412	-
berB1.1	1,000	1,000	1,000	1,000	1,000	1,000
berC1.1	1,000	1,000	0,111	0,514	0,706	1,000
berC1.2	-	-	0,667	0,358	0,294	-
berC2.2	-	-	0,222	0,128	-	-
berD1.1	1,000	1,000	1,000	1,000	1,000	1,000
berE1.1	1,000	0,857	0,667	0,949	0,928	0,983
berE1.2	-	0,071	-	-	-	-
berE1.3	-	0,071	0,333	0,051	0,072	-
berE1.4	-	-	-		-	0,017
berF1.1	0,491	abs†	1,000	1,000	1,000	-
berF2.2	0,130	abs	-	-	-	0,983
berF1.2	0,379	0,357	-	-	-	-
berF2.3	-	0,071	-	-	-	-
berF2.4	-	-	-	-	-	0,017
berG1.1	1,000	1,000	1,000	1,000	1,000	0,150
berG2.2	-	-	-	-	-	0,350
berG1.2	-	-	-	-	-	0,383
berG1.3	-	-	-	-	-	0,017
berG2.3	-	-	-	-	-	0,100
Number of zygotic combinations	10	abs	11	12	11	13
% of heterozygous larva	abs	85,7	78	82,1	59	51,7
Number of heterozygous inversions per specimen	0,480	0,643	1,110	1,000	0,650	0,533
Number of inversions per arm	0,29	0,71	0,43	0,43	0,43	0,71

† abs - data are absent.

**Arm B** was monomorphic. Banding sequence berB1 remain unmapped due to the complex rearrangements that differ the banding pattern in the arm B of *Chironomus bernensis* from the standard one of *Chironomus piger*.

**Figure 4.**
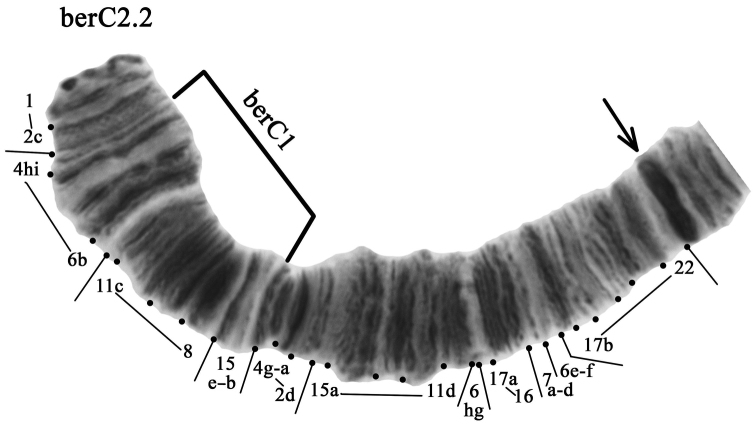
Homozygous zygotic combination berC2.2. The designations are the same as in Fig. 2.

**Arm C** has two banding sequences – berC1 and berC2. The banding sequence berC1 was dominant in all studied populations (Table 3, 4). The banding sequence berC2 is new for the species and described for the first time (Fig. 4, Table 2–4). It differs from berC1 by one simple inversion step that involves regions 4hi-6b 11c-8 15e-b: berC2 1-2c 4hi-6b 11c-8 15e-b 4g-2d 15a-11d 6gh 17a-16 7d-a 6f-c 17b-22 C

The banding sequence berC2 was found in studied populations with high frequency in both homozygous and heterozygous state (Table 3, 4).

**Arm D** is monomorphic with banding sequence berD1 found in homozygote state (Fig. 2, Table 2–4).

**Figure 5.**
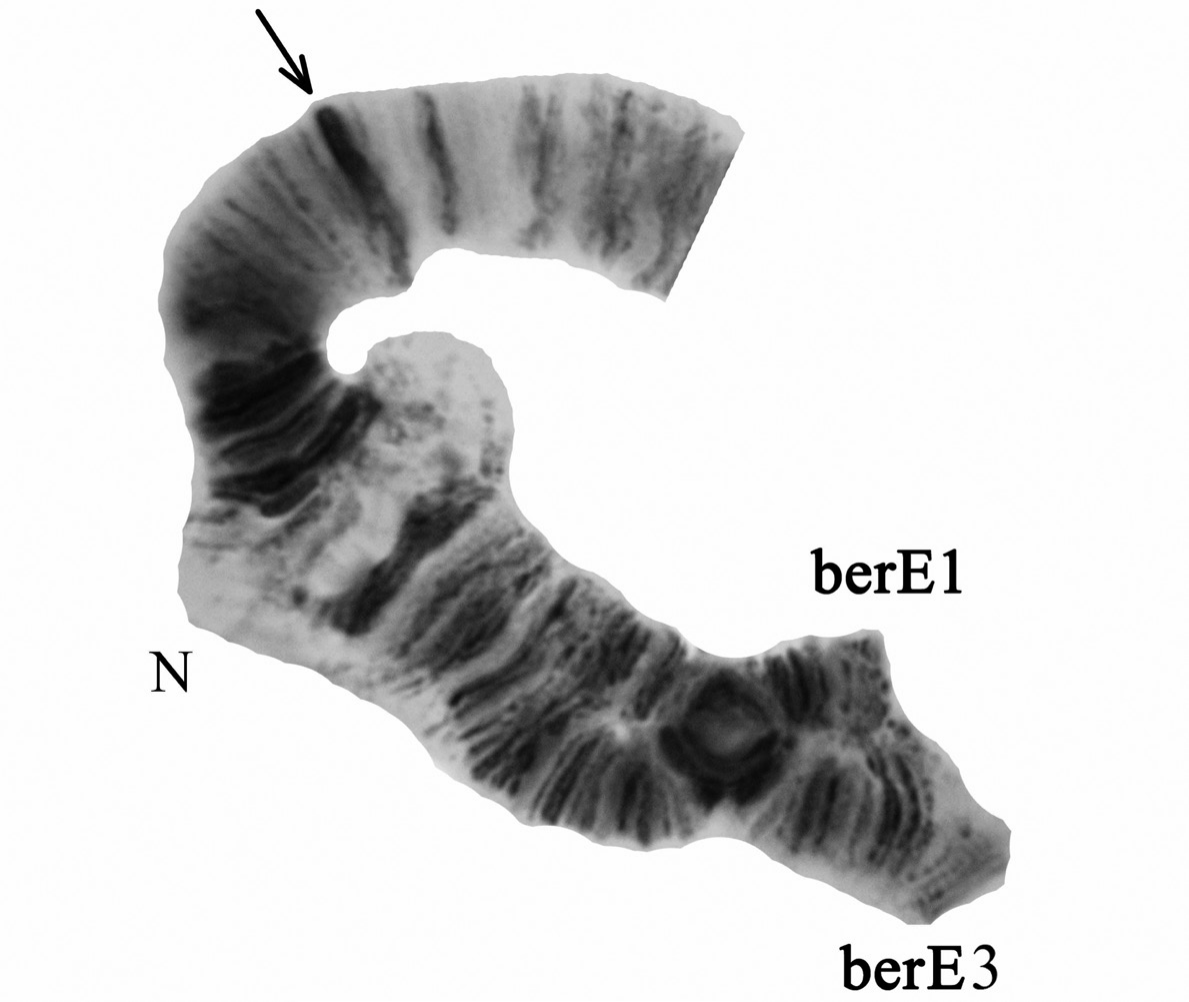
Heterozygote berE1.3 The designations are the same as in Fig. 2.

**Arm E** had two banding sequences–berE1 and berE3 (Table 2–4). The banding sequence berE1 was dominant in all studied North Caucasian populations (Table 3, 4). The banding sequence berE3 has been found only in heterozygous state (Fig. 5, Table 3, 4).

**Arms F** and **G** were monomorphic and presented by sequences berF1 and berG1, respectively (Fig. 2, Table 2–4).

In all three North Caucasian populations the number of banding sequences was identical and equal to 10 (Table 3). The number of zygotic combinations found in studied populations varied from 11 to 12 (Table 4). From 59 to 82% of larvae were heterozygous (Table 4).

In total, 12 genotypic combinations have been found (Table 5). Each studied population was characterized by different dominant genotypic combination. Thus, in RKB (the former riverbed in the mouth of the Cherek River) dominant genotypic combinations were berA2.2B1.1C1.2D1.1E1.1F1.1G1.1 and berA2.2B1.1C1.2D1.1E1.3F1.1G1.1, in RKB (in the vicinity of Zhemtala village, long-term water body) – berA1.2B1.1C1.1D1.1E1.1F1.1G1.1; in KCR (Malyi Zelenchuk River) – berA1.2B1.1C1.1D1.1E1.1F1.1G1.1 and berA2.2B1.1C1.1D1.1E1.1F1.1G1.1.

**Table 5.** Genotypic combinations *Chironomus bernensis* from Central Caucasus and Ciscaucasia.

Genotypic combinations	RKB, former riverbed in mouth of Cherek River (original data) 9 larvae	RKB, near Zhemtala village, long-term pool (original data) 39 larvae	KCR, M. Zelenchuk River (original data) 17 larvae
A1.1B1.1C1.1D1.1E1.1F1.1G1.1	0	0	0,176
A1.1B1.1C1.2D1.1E1.1F1.1G1.1	0	0,025	0
A1.1B1.1C1.1D1.1E1.3F1.1G1.1	0	0	0
A1.1B1.1C2.2D1.1E1.3F1.1G1.1	0	0	0,059
A1.2B1.1C1.1D1.1E1.1F1.1G1.1	0,111	0,308	0,235
A1.2B1.1C1.2D1.1E1.1F1.1G1.1	0	0,128	0,059
A1.2B1.1C1.1D1.1E1.3F1.1G1.1	0	0,025	0,059
A1.2B1.1C2.2D1.1E1.1F1.1G1.1	0	0,103	0
A1.2B1.1C1.1D1.1E1.3F1.1G1.1	0	0	0
A1.2B1.1C1.2D1.1E1.3F1.1G1.1	0	0,025	0
A2.2B1.1C1.1D1.1E1.1F1.1G1.1	0	0,179	0,235
A2.2B1.1C1.2D1.1E1.1F1.1G1.1	0,333	0,179	0,117
A2.2B1.1C1.2D1.1E1.3F1.1G1.1	0,333	0	0
A2.2B1.1C2.2D1.1E1.1F1.1G1.1	0,222	0,025	0
number of genotypic combinations	4	9	7

### Comparison of chromosomal polymorphism of *Chironomus bernensis* from the Central Caucasus and Ciscaucasia and other parts of the range

**Figure 6.**
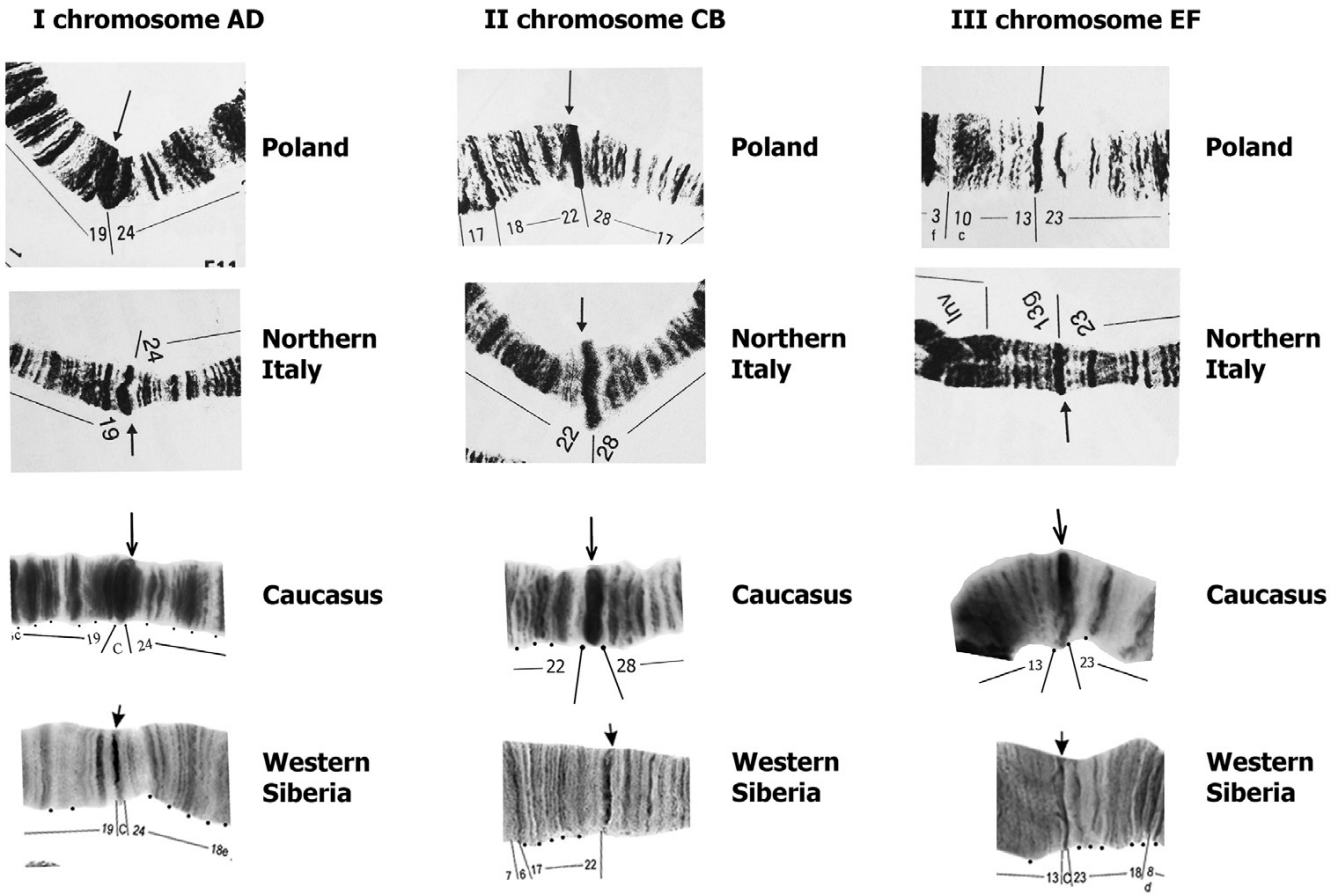
Comparison of pericentromeric regions of polytene chromosomes of *Chironomus bernensis* from Caucasian, European and Siberian populations.

As stated above, in all the long polytene chromosomes of *Chironomus bernensis* from the studied North Caucasian populations the centromere bands are large and belong to n-type according to the classification by Shobanov (2002) (Fig. 6). In Siberian populations (Istomina and Kiknadze 2004, Kiknadze et al. 2007) and in the photo of chromosomes in the first description of *Chironomus bernensis* from Swiss populations (Wülker and Klötzli 1973), the centromere bands are thin and belong to s-type. The large centromeric bands of this species were found in the populations of Bulgaria, Poland, Northern Italy (Michailova 1989, Michailova et al. 2002, Petrova and Michailova 2002).

Data for Polish and Italian populations are presented on the basis of publications of Michailova (1989); Michailova and coauthors (Michailova et al. 2002), Petrova and Michailova (2002), data for Siberian populations are presented on the basis of publications of Istomina and Kiknadze (2004), Kiknadze and coauthors (Kiknadze et al. 2007).

Unfortunately, because of the low number of specimens of *Chironomus bernensis* found in most populations of Central Caucasus and Ciscaucasia water bodies studied, only three populations with a significant number of larvae – the former riverbed in the mouth of the Cherek River near Oktyabrskaya village, the long-term water body near Zhemtala village, the backwater in the main riverbed of Malyi Zelenchuk River near Adyl-Khalk village – were used for comparison with populations from other geographic regions (Table 3, 4).

**Arm A.** The populations from the North Caucasus, as well as populations from Europe–Switzerland (Wülker and Klötzli 1973) and Italy (Petrova and Michailova 2002) – are characterized by the presence of two banding sequences in this arm, berA1 and berA2 (Table 3, 4), whereas only berA1 was present in populations of Western Siberia (Istomina and Kiknadze 2004). At the same time it should be noted that populations from the North Caucasus and Europe differ significantly by the frequencies of banding sequence berA1 and berA2: while the former was dominant in Western Europe, the latter dominated in North Caucasian populations, occurring there in both the heterozygote and homozygote state.

**Arm B and D** of *Chironomus bernensis* were monomorphic in all studied populations.

**Arm C** of *Chironomus bernensis* were monomorphic in populations from Europe and Siberia but showed high level of inversion polymorphism in studied Caucasian populations due to the presence of a new banding sequence berC2 that might be endemic for this region. However, for *Chironomus bernensis* from Spain unmapped chromosomal rearrangement in the arm C was early indicated (Real et al. 2000). The high frequencies of heterozygotes berC1.2 and homozygotes berC2.2 in Caucasian populations (Table 3, 4) clearly distinguishes them from all other populations.

In the **arm E** all studied populations of *Chironomus bernensis* share the same dominant banding sequence berE1. At the same time populations from all regions differ from each other by sets of additional banding sequences found in heterozygote state. Thus, in Switzerland this arm was completely monomorphic (Wülker and Klötzli 1973), in Italy two banding sequences – berE2 and berE3 (Petrova and Michailova 2002) – were found with low frequencies in heterozygotes with berE1, while only heterozygotes berE1.3 were found in Caucasian populations and berE1.4 – in populations from Western Siberia (Istomina and Kiknadze 2004). The comparison of the inversion banding sequences of the arm E from different populations shows the most similarity between Caucasian and Italian populations.

**Arm F** of *Chironomus bernensis* in Caucasian populations was monomorphic and presented only by the standard banding sequence berF1 unlike the populations from other regions. In the population of Switzerland (Wülker and Klötzli 1973) the approximately equal number of homo- (ber F1.1) and heterozygotes (ber F1.2) was observed. In the Siberian population banding sequence berF2 was strictly dominant with the only other banding sequence being berF4 that was present with a low frequency in a heterozygote state (berF2.4) (Istomina and Kiknadze 2004), which clearly distinguishes the Siberian population of *Chironomus bernensis*.

**Arm G** of *Chironomus bernensis* was monomorphic in both European and Caucasian populations and was presented by the standard banding sequence berG1. At the same time in the Siberian population three banding sequences were found in different zygotic combination (Istomina and Kiknadze 2004) with berG1.2 being the dominant one, which clearly distinguishes this population from the other ones.

Thus, summarizing all data it can be concluded that a significant degree of divergence can be seen between populations of Europe, Caucasus and Western Siberia.

The inversion polymorphism of populations of *Chironomus bernensis* from the North Caucasus has much higher level of heterozygous inversions per specimen in comparison with the early studied populations, i.e. 0,65 to 1,11 (Tables 3–5). In the number of genotypic combinations (11), number of banding sequences per population (10) and number of inversions per arm (0,43), the Caucasian populations of this species are intermediate between European (respectively: 10, 9 and 0,29) and Siberian (respectively: 13, 11 and 0,71) populations.

**Figure 7.**
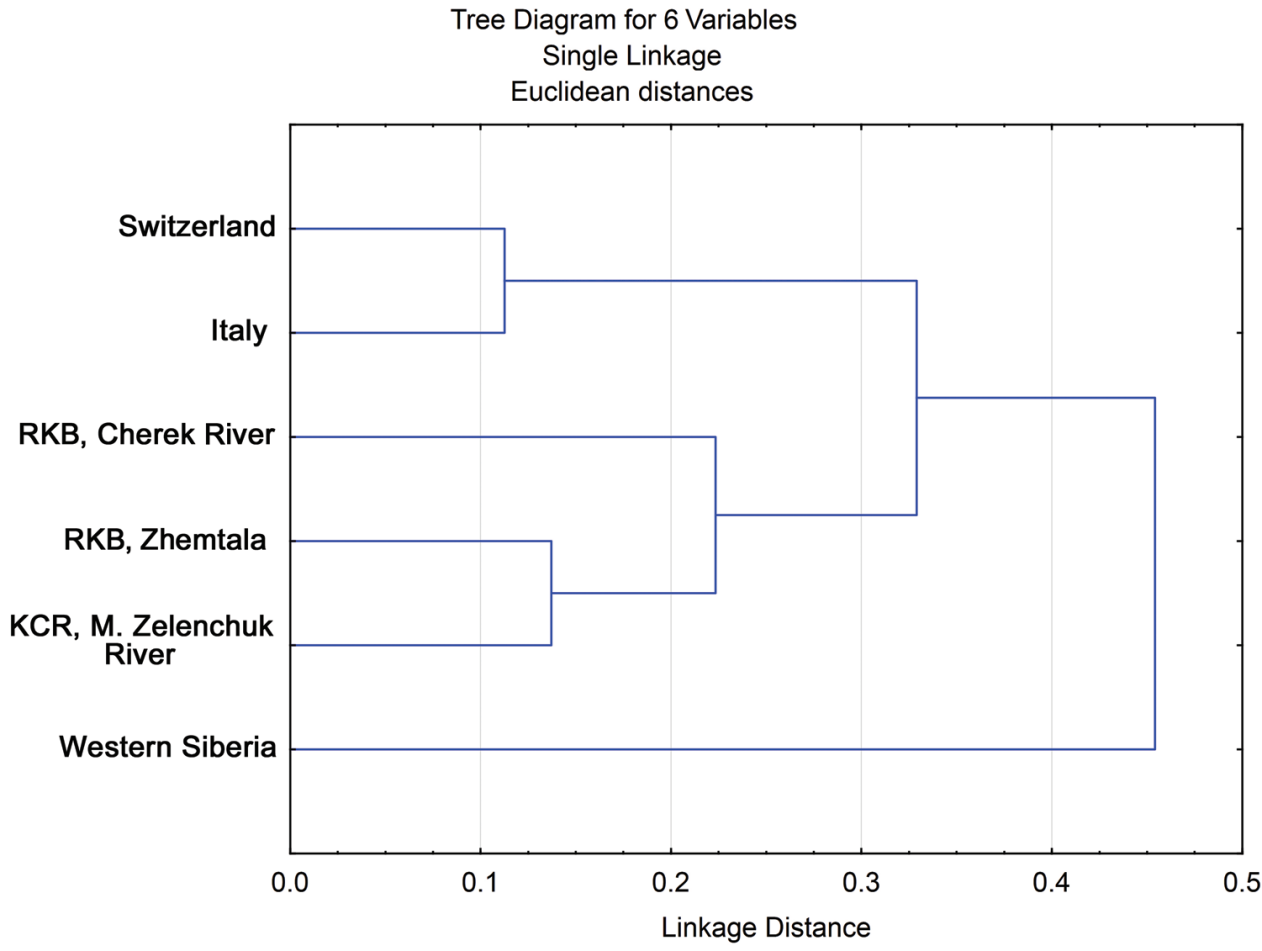
The dendrogram of cytogenetic distances between the samples from different populations of *Chironomus bernensis*.

Cytogenetic distances (Table 6), was measured by Nei criteria (1972) on basis of the original data and data of other authors on inversion polymorphism of the species in Europe and Siberia (Fig. 7). These distances indicate the significant distance of the Siberian populations of *Chironomus bernensis* and of intermediate position of the Caucasian populations between the populations of Western Europe and Western Siberia.

**Table 6.** Value of cytogenetic distances between the different populations of *Chironomus bernensis*.

Population	Switzerland	Italy	RKB (Cherek river)	RKB (Zhemtala)	KCR (M. Zelenchuk River)	Western Siberia
Switzerland	0					
Italy	0,054	0				
RKB (Cherek River)	0,343	0,409	0			
RKB (Zhemtala)	0,176	0,206	0,082	0		
KCR (M. Zelenchuk River)	0,111	0,159	0,092	0,015	0	
Western Siberia	0,130	0,142	0,645	0,424	0,322	0

The dendrogramm was constructed on the basis of Nei criteria (1972) using NJ- method.

In establishing of cytogenetic distances for populations of Siberia, Switzerland and Italy data of other authors were used (Wülker and Klötzli 1973, Petrova and Michailova 2002, Istomina and Kiknadze 2004).

## Discussion

In the Central Caucasus (the northern macroslope) and Ciscaucasia *Chironomus bernensis* has been found for the first time. At present, 17 banding sequences including berC2 are known in the banding sequences pool of *Chironomus bernensis*. The comparative analysis of chromosomal polymorphism between the Caucasian populations and populations of other regions has revealed specific peculiarities: the presence of sequence berA2 in homozygous state, which was not registered in the populations studied earlier, and the presence of banding sequence berC2, which is probably endemic for the region.

The morphological characteristics such as the number of premandible teeth are diagnostic for *Chironomus* species. Thus, among the species of this genus more than two teeth of the premandible can be found in larvae dwelling in the brackish water bodies, i.e. *Chironomus behningi* Goetgh. with five teeth (Pankratova 1983, Polukonova and Beljianina 2002); *Chironomus albidus* Konst. (Konstantinov 1956) and *Chironomus* sp. (sibling species of *Chironomus albidus*, apparently belonging to *Chironomus paraalbidus* Beljanina et al. 2005a) with three teeth (Polukonova et al. 2004, Beljianina et al. 2005b, Polukonova 2007). It can be suggested that this morphological peculiarity emerged due to such special feature of the chemical composition in water bodies of the Caucasus as increased mineralization. However, such an assertion needs additional research on the water mineralization level in the collection sites of *Chironomus bernensis* in the Central Caucasus (the northern macroslope) and Ciscaucasia.

The other significant diagnostic characteristic that allows differentiating the species of genus *Chironomus* is the centromere type (Shobanov 2000, 2002). Thus, several pairs of sibling species with identical banding sequences in the polytene chromosomes (homosequential species), such as *Chironomus piger* and *Chironomus riparius* (Keyl and Strenzke 1965, Polukonova et al. 1996, Karmokov et al. 2011) or *Chironomus nuditarsis* and *Chironomus curabilis* (Polukonova et al. 2003, 2005, Polukonova 2005a, b), were found to be different in the size of the pericentromeric heterochromatin. Although it is necessary to note that intra- and interpopulation chromosomal polymorphism can be observed for this characteristic (Iliynskaya 1984, Kiknadze and Siirin 1991, Kiknadze et al. 1991b), which can complicate its use as a species-specific criteria especially in the cases when the difference in centromere size of different species is not very significant.

The dominance of different genotypic combinations at various sites of the Caucasus probably can be explained by the fact that in some areas some combinations can be more adaptive than the others. Perhaps this is happening due to a different level of mineralization, temperature and degree of eutrophication in the different collection sites.

Caucasian populations on the dendrogram occupy an intermediate position between Italian and Swiss populations, on the one hand, and Western Siberian population, on the other. Such arrangement agrees rather well with the geographic location of the studied regions and may reflect the true course of settlement of the species (either from west to east or from east to west). For more specific allegations more researches are needed.

In the context of the data mentioned above, further researches on *Chironomus bernensis* from geographically distant regions are necessary, as there is possibility that the presently known species is actually polytypic and consists of several sibling species.

## References

[B1] Beljanina SI Polukonova NV Zinchenko TD (2005a) Karyotype and morphology of larva of non-biting midge of the genus *Chironomus* from Caspian Sea. Tsitologiia 47(4): 331–337. [In Russian] 16706156

[B2] Beljanina SI Polukonova NV Zinchenko TD (2005b) New species of non-biting midges of genus *Chironomus paraalbidus*, sp. n. (Chironomidae, Diptera) from Caspian Sea. Zoologichesky Zhurnal 85(4): 65–71. [In Russian with English summary]

[B3] Dévai Gy Miskolczi M Wülker W (1989) Standardization of chromosome arms B, C, and D in *Chironomus* (Diptera, Chironomidae). Advances in Chironomidology: Acta Biologica Debricina. Supplementum oecologica Hungarica 2(1): 79–92.

[B4] Dyomin SYu Iliynskaya NB (1988) Change of the density of polytene chromosomes from different organs of *Chironomus plumosus* larvae. Tsitologiia 30(4): 407–415. [In Russian]

[B5] Dyomin SYu Shobanov NA (1990) Karyotype of *Chironomus entis* from the plumosus group (Diptera, Chironomidae) living in the European part of the Soviet Union. Tsitologiia 32(10): 1046–1054. [In Russian]

[B6] Iliynskaya NB (1984) Polytene chromosome characteristics with different degree of compactness in larvae of *Chironomus* from nature populations. Tsitologiia 26(5): 543–551. [In Russian]

[B7] Istomina AG Kiknadze II (2004) *Chironomus bernensis* Klotzli, 1973 (Diptera, Chironomidae) in West Siberia: kayotype and chromosomal polymorphism. Eurasian entomological journal 3(4): 283–288. [In Russian]

[B8] Karmokov MKh Khatukhov AM Polukonova NV (2011) Comparison of two closely related species of *Chironomus* – *Ch. riparius* Meigen (1818) and *Ch. piger* Strenzke (1959) (Diptera, Chironomidae) from Central Caucasus and Lower Volga region by ecological and geographical peculiarities, morphological characters of larva and karyotype. Collection of scientific papers of Academy of Science of Chechen Republic, Grozny 3: 206–219. [In Russian]

[B9] Keyl H-G (1962) Chromosomenevolution bei *Chironomus*. II. Chromosomenumbauten und phylogenetische Beziehungen der Arten. Chromosoma 13(4): 464–514. doi: 10.1007/BF00327342

[B10] Keyl H-G Strenzke K (1965) Taxonomie und Cytologie von zwei Subspezies der Art *Chironomus thummi*. Naturforsen 11b: 727–735.

[B11] Kiknadze II Shilova AI Kerkis IE Shobanov NA Zelentsov II Grebenyuk LP Istomina AG Prasolov VA (1991) Karyotypes and larval morphology in the tribe Chironomini. Atlas. Novosibirsk, 113 pp [In Russian with English summary]

[B12] Kiknadze II Siirin MT (1991) The polymorphism of pericentromeric heterochromatin in *Chironomus plumosus* L. Tsitologiia 33(3): 60–68. [In Russian]

[B13] Kiknadze II Siirin MT Filippova MA Gunderina LI Kalachikov SM (1991) The change of the pericentromeric heterochromatin mass is one of important ways of the chironomid evolution. Tsitologiia 33(12): 90–98. [In Russian]1726546

[B14] Kiknadze II Gunderina LI Butler MG Wülker W Martin J (2007) Chromosomes and continents. VOGiS Herald 11(2): 332–351. [In Russian with English summary]

[B15] Konstantinov AS (1956) On systematics of genus *Chironomus* Meig. Trudi Saratovskogo otdeleniia VNIORK 4: 155–191. [In Russian]

[B16] Laville H (1971) Recheches sur les Chironomides (Diptera) lacustres du massif de Neovielle. Premiere partie: Sistematique, ecologie, phenologie. Annales de Limnologie 7: 173–332. doi: 10.1051/limn/1971006

[B17] Michailova P (1989) The polytene chromosomes and their significance to the systematics of the family Chironomidae, Diptera. Acta Zoologica Fennica 189: 1–107.

[B18] Michailova P Krastanov B Kownacki A (2002) Cytotaxonomical characteristics of genus *Chironomus* Meigen (Diptera, Chironomidae) from different localities of Poland. Annales Zoologici (Warszawa) 52(2): 215–225.

[B19] Michailova P Szarek-Gwiazda E Kownacki A (2009) Effect of contaminants on the genome of some species of genus *Chironomus* (Chironomidae, Diptera) live in sediments of Dunajec River and Czorsztyn Reservoir. Water, Air & Soil Pollution 202: 245–258. doi: 10.1007/s11270-008-9973-8

[B20] Nei M (1972) The genetic distance between populations. American Naturalist 106: 283–292. doi: 10.1086/282771

[B21] Pankratova VYa (1983) Larvae and pupae of the subfamily Chrinominae of the USSR fauna (Diptera, Chironomidae - Tendipedidae). Leningrad, 296 pp [In Russian]

[B22] Petrova NA Michailova P (2002) Cytogenetic characteristics of *Chironomus bernensis* Klotzli (Diptera: Chironomidae) from a heavy metal polluted stations in North Italy. Annales Zoologici (Warszawa) 52(2): 227–233. doi: 10.1023/A:1021687722053

[B23] Polukonova NV Beljanina SI Durnova NA (1996) Differential diagnosis of homosequent species *Chironomus piger* Strenzke and *Ch. riparius* Meigen. In: Ecology, evolution and systematic of chironomids. Inst. Ecol. Volga Basin and Inst. Biol. Inland Waters, Tolyatti, Borok, 109–115. [In Russian with English summary]

[B24] Polukonova NV Beljanina SI (2002) On the Possibility of Hybridogenesis in the Origin of Midge *Chironomus usenicus* Loginova et Beljanina (Chironomidae, Diptera). Russian Journal of Genetics 12: 1385–1390. [In Russian with English summary] 12575448

[B25] Polukonova NV Beljanina SI Michailova PV (2003) Morpho-karyotipic approach to solving the discussion questions of systematic on an example of *Chironomus curabilis* Beljanina, Sigareva, Loginova, 1990 (Diptera, Chironomidae). In: Evolution problems. Collection of scientific papers. Vladivostok 5: 207–212. [In Russian with English summary]

[B26] Polukonova NV Beljanina SI Zinchenko TD Dairova D (2004) New data on the chironomids fauna (Chironomidae, Diptera) of Caspian Sea and its basin. Ecological and biological problems of Caspian Sea basin. Proceeding of VII All-Russian scientific conference. Astrakhan, 91–92. [In Russian]

[B27] Polukonova NV (2005a) A comparative morphological analysis of the midges *Chironomus nuditarsis* and *Ch. curabilis* (Chironomidae, Diptera). 1. Preimaginal stages. Zoologichesky Zhurnal 84(3): 367–370. [In Russian with English summary]

[B28] Polukonova NV (2005b) A comparative morphological analysis of the midges *Chironomus nuditarsis* and *Ch. curabilis* (Chironomidae, Diptera). 2. Midge males and females. Zoologichesky Zhurnal 84(3): 371–376. [In Russian with English summary]

[B29] Polukonova NV (2005c) Morphological and chromosomal differentiation of non-biting midges (Chironomidae, Diptera) in the process of speciation. Doctoral Dissertation, Saratov State Medical University named after V.I. Razumovsky, Saratov, Russian Federation, 564 pp [In Russian]

[B30] Polukonova NV Beljanina SI Zinchenko TD (2005a) New species of non-biting midges of genus *Chironomus paraalbidus*, sp. n. (Chironomidae, Diptera) from Caspian Sea. Zoologichesky Zhurnal 85(4): 65–71. [In Russian with English summary]

[B31] Polukonova NV Beljanina SI Michailova PV Goligina VV (2005b) Comparative analysis of the midges *Chironomus nuditarsis* and *Ch. curabilis* karyoforms and karyofunds (Chironomidae, Diptera). Zoologichesky Zhurnal 84(2): 195–206. [In Russian with English summary]

[B32] Polukonova NV (2007) Discussion about status of saltish water *Chironomus albidus* Konst and *Ch. paraalbidus* Beljanina et al. 2004 in system of genus *Chironomus* Meigen 1803 (Chironomidae, Diptera). Entomological and parasitologic researches of the Volga region. Saratov 6: 81–84. [In Russian with English summary]

[B33] Real M Rieradevall M Prat N (2000) *Chironomus* species (Diptera: Chironomidae) in the profundal benthos of Spanish reservoirs and lakes: factors affecting distribution patterns. Freshwater Biology 43(1): 1–18. doi: 10.1046/j.1365-2427.2000.00508.x

[B34] Shobanov NA (2000) The genus *Chironomus* Meigen (Diptera, Chironomidae). Taxonomy, Biology, and Evolution. Doctoral Dissertation, Zoological Institute, Russian Academy of Sciences, St. Petersburg, Russian Federation, 464 pp [In Russian]

[B35] Shobanov NA (2002) Evolution of the genus *Chironomus* (Diptera, Chironomidae). 1. Ancestral form and major lines of phylogenesis. Zoologichesky Zhurnal 81(4): 463–468. [In Russian with English summary]

[B36] Sokolov VE Tembotov AK (1989) Mammals of Caucasus: Insectivores. Moscow, 548 pp [In Russian with English summary]

[B37] Wülker W Klötzli AM (1973) Revision der Gattung *Chironomus* Meig. IV. Arten des lacunarius- (commutatus-) complexes. Archive Hydrobiologia 72(2): 474–489.

